# Association of soluble suppression of tumorigenicity 2 with mortality and adverse outcomes in chronic kidney disease: a systematic review and meta-analysis

**DOI:** 10.1007/s10157-024-02506-6

**Published:** 2024-04-27

**Authors:** Ioannis Bellos, Smaragdi Marinaki, Pagona Lagiou, Vassiliki Benetou

**Affiliations:** 1 Department of Hygiene, Epidemiology and Medical Statistics, Medical School, National and Kapodistrian University of Athens, 75, Mikras Asias Str, 115 27 Athens, Greece; 2 Department of Nephrology and Renal Transplantation, Medical School, Laiko General Hospital, National and Kapodistrian University of Athens, Athens, Greece

**Keywords:** ST2, Chronic kidney disease, Prognosis, Cardiovascular, Survival

## Abstract

**Background:**

Early risk stratification is necessary to prevent chronic kidney disease progression and complications. This systematic review aims to evaluate the association of soluble suppression of tumorigenicity 2 (sST2), a member of the interleukin-1 receptor family, with all-cause mortality, cardiovascular disease and renal function deterioration among chronic kidney disease patients.

**Methods:**

PubMed, Scopus, Web of Science, CENTRAL and Google Scholar were systematically searched from inception to December 20, 2023. Cohort studies examining the prognostic role of sST2 levels in pre-dialysis and dialysis patients were included. In case of 3 or more studies per outcome, conventional and dose–response meta-analyses were conducted.

**Results:**

Overall, 21 studies were included comprising 15,100 patients. In pre-dialysis patients, the qualitative synthesis of studies suggested that high sST2 is associated with significantly increased all-cause mortality, while evidence regarding cardiovascular events or kidney disease progression was conflicting. In the dialysis population, high sST2 was linked to an elevated risk of all-cause (Hazard ratio-HR: 3.00, 95% confidence intervals-CI: 1.95–4.61) and cardiovascular (HR: 2.38, 95% CI: 1.69–3.34) mortality. Dose–response meta-analysis suggested a log-linear association of sST2 with both all-cause (*χ*^*2*^: 34.65, *p value* < 0.001) and cardiovascular (*χ*^*2*^: 29.14, *p value* < 0.001) mortality, whereas findings regarding cardiovascular events were limited with mixed results.

**Conclusions:**

High sST2 values are associated with an increased risk of all-cause mortality in pre-dialysis and dialysis patients, as well as with an elevated risk of cardiovascular mortality in the dialysis population. Further studies are needed to elucidate its potential association with cardiovascular events and kidney disease progression.

## Introduction

Chronic kidney disease constitutes a major public health concern, associated with high morbidity and mortality rates. Its incidence is rising, affecting more than 10% of the global population [1]. It represents an important cause of death and by 2040 it is estimated to be the 5th leading cause of years of life lost worldwide [2]. Cardiovascular disease is the main complication of chronic kidney disease due to the presence of traditional risk factors, especially diabetes mellitus, hypertension and metabolic syndrome along with the existence of renal disease-specific factors, such as the accumulation of uremic toxins, inflammation and vascular calcification [3]. In this context, patients with renal impairment are at increased risk of developing atherosclerotic cardiovascular disease, heart failure, valvular heart disease, as well as sudden cardiac death [4, 5]. Early identification of patients at high risk of complications may enable the prompt implementation of targeted interventions aiming for cardiovascular protection and prevention of progression to kidney failure [6]. Several biomarkers, such as N-terminal pro b-type natriuretic peptide [7], galectin-3 [8] and growth/differentiation factor-5 [9] have been proposed to enhance the risk stratification of chronic kidney disease patients, although the optimal screening strategy remains still under investigation.

Suppression of tumorigenicity 2 (ST2), a member of the interleukin-1 receptor family, has recently gained interest as a candidate biomarker of cardiovascular disease outcomes. It serves as the receptor of interleukin-33 and is present in a transmembrane and a soluble (sST2) isoform [10]. The interplay of transmembrane ST2 with interleukin-33 has been shown to exert cardioprotective effects through the inhibition of myocardial fibrosis, hypertrophy and apoptosis [11]. On the other hand, sST2 serves as a decoy receptor which avidly binds to interleukin-33 and competes with transmembrane ST2, eliminating thus the aforementioned cardioprotection [12]. As a result, high sST2 values have been associated with hypertension and diabetes mellitus in the general population and have been suggested to effectively predict adverse outcomes in patients with acute coronary syndrome [13] and heart failure [14, 15].

The interleukin-33/ST2 axis has been implicated in the development of renal fibrosis [16], being involved in the pathogenesis of acute kidney injury, diabetic nephropathy and chronic kidney disease [17]. However, the exact prognostic value of sST2 levels in patients with chronic kidney disease remains currently unclear. The present systematic review and meta-analysis aims to gather the available literature knowledge in the field and shed more light on the potential association of sST2 with the risk of mortality, cardiovascular disease and renal function deterioration among patients with chronic kidney disease.

## Materials and methods

### Study design

This systematic review was reported following the PRISMA (Preferred Reporting Items for Systematic Reviews and Meta-Analyses) guidelines [18]. The study protocol has been prospectively registered and is publicly available (dx.doi.org/10.17504/protocols.io.dm6gp3m48vzp/v1). No ethical approval was required as already published data were used.

### Eligibility criteria

The population of the study consisted of adults diagnosed with chronic kidney disease. Both pre-dialysis and dialysis (hemodialysis or peritoneal dialysis) patients were included. Kidney transplant recipients were excluded. The exposure of interest was circulating sST2 levels. The primary outcome of interest was all-cause mortality. Secondary outcomes included kidney disease progression, cardiovascular mortality, major adverse cardiovascular events (MACE) and incident heart failure. Both prospective and retrospective cohort studies were held potentially eligible. Case–control, descriptive, cross-sectional, animal and in vitro studies, as well as case reports/series and review articles were excluded. Chronic kidney disease stages were defined following the KDIGO (Kidney Disease: Improving Global Outcomes) guidelines [19].

### Literature search

Literature search was performed by systematically searching PubMed, Scopus, Web of Science and CENTRAL (Cochrane Central Register of Controlled Trials). In addition, Google Scholar was screened to provide grey literature coverage, while the full reference lists of the included studies were inspected to recognize potential missing articles (“*snowball*” method [20]). No date/language restrictions were applied. All databases were searched from inception till December 20,2023. The search was conducted using a combination of MeSH (Medical Subject Headings) terms and key-words. The main search algorithm was the following: “(Suppression of Tumorigenicity OR st2 OR sst2) AND (“Renal Insufficiency, Chronic” [Mesh] OR “chronic kidney disease” OR “CKD” OR “kidney disease” OR “renal disease” OR “kidney failure” OR “renal failure” OR “kidney insufficiency” OR “renal insufficiency” OR nephropathy)”.

### Study selection

The process of study selection followed three consecutive stages. Firstly, the titles and abstracts of all electronical articles were screened to assess for eligibility. Subsequently, all articles that were considered as potentially eligible were retrieved in full-text form. Then, the studies that did not report the outcomes of interest or met any of the exclusion criteria were excluded. The selection of the included studies was performed by two researchers independently, resolving any discrepancies after discussion with all authors.

### Data extraction

The following data were extracted from the included studies using pre-piloted forms: year of publication, country, eligibility criteria, sample size, study design, type of population, sST2 assay, participants’ age, sex, percentage of hypertension, diabetes mellitus, estimated glomerular filtration rate, body mass index, dialysis vintage, history of cardiovascular disease, as well as the necessary information regarding the outcomes of interest. All data were extracted by two authors independently and any discrepancies were resolved through their consensus.

### Quality assessment

The risk of bias of the included studies was evaluated using the ROBINS-I tool [21], adjusted for exposure studies, taking into consideration the following domains: confounding, selection of participants, classification of exposures, departures from intended exposures, missing data, measurement of outcomes and selection of the reported results. The risk of bias evaluation was performed by two researchers independently, resolving any disagreements through the consensus of all authors.

### Data analysis

All outcomes were initially evaluated qualitatively. Pre-piloted forms were used to capture the necessary information regarding the outcomes of interest. Pre-dialysis and dialysis patients were separately assessed. Circulating sST2 levels could be evaluated as a continuous variable or as a binary one in case cut-off values were introduced in the included studies. For time-to-event endpoints, hazard ratios (HR) were extracted along their 95% confidence intervals (CI). Statistical significance was defined by the two-sided *p value* threshold of 0.05. Meta-analysis was performed in case of at least 3 studies per outcome were included. In meta-analysis, circulating sST2 was treated only as a binary variable, using the cut-offs that were introduced by the original studies. Conventional meta-analysis was conducted by comparing the highest to the lowest sST2 category. Random-effects statistical models were fitted due to the high expected methodological heterogeneity, using the restricted maximum likelihood method. To account for the small number of studies, the Knapp-Hartung adjustment [22] was applied as a sensitivity analysis. The statistical inter-study heterogeneity was quantified by the inconsistency index (*I*^*2*^), with values above 50% indicating remarkable statistical heterogeneity [23]. The 95% prediction intervals were calculated to provide estimates of the effects to be expected by future studies in the field [24]. Publication bias was planned to be statistically tested in case of 10 or more studies per outcome [25]. Dose–response meta-analysis was also conducted to define the potential exposure–response relationship between sST2 levels and mortality risk. In particular, a non-linear model using restricted cubic splines was applied in a one-stage approach [26]. Restricted cubic splines were located at the 25th, 50th and 75th percentiles of the sST2 level distribution. Statistical analysis was conducted in R-4.0.4 (“*metafor*” [27] and “*dosresmeta*” [28] packages).

## Results

### Study selection

**Fig. 1:**
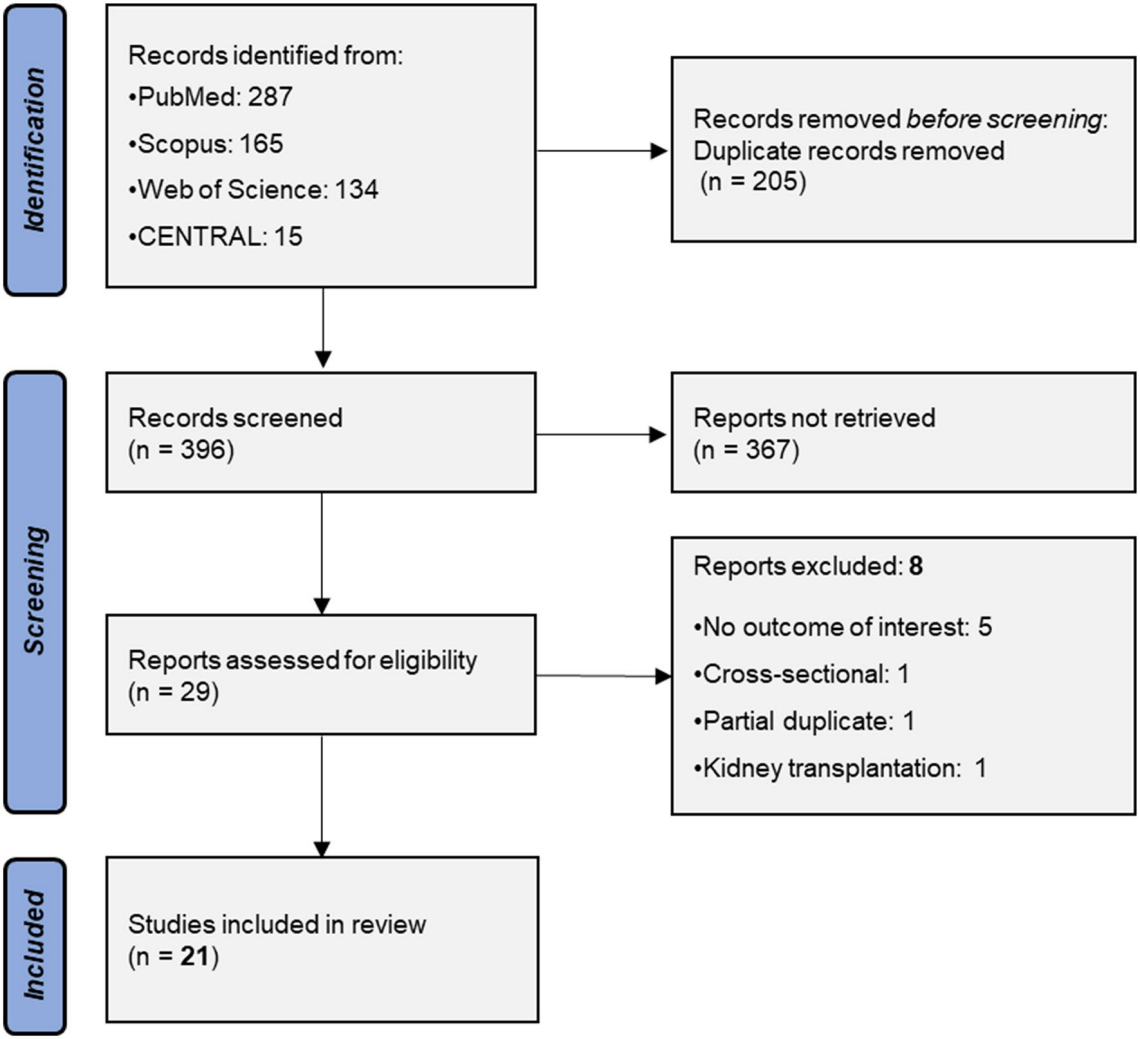
Search plot PRISMA flowchart

Figure 1 depicts the process of study selection in a PRISMA flowchart. Database search resulted in 601 records. After deduplication, 396 articles were screened and a cohort of 29 studies were retrieved in full text. Of them, 8 studies were excluded for the following reasons: no outcome of interest (n = 5) [29–33], cross-sectional design (n = 1) [34], partial duplicate of a study already included (n = 1) [35] and evaluation of kidney transplant recipients (n = 1) [36]. As a result, 21 studies [37–57] were finally included, comprising a total of 15,100 (12,098 pre-dialysis and 3,002 dialysis) patients.

### Included studies

The methodological characteristics of the included studies are presented in Table 1. Eight studies were conducted in the United States of America, 5 in Europe and 8 in Asia. Nineteen studies adopted a prospective design, while 2 studies were retrospective cohort ones. Dialysis patients were evaluated in 9 studies, with hemodialysis patients being exclusively included in 8 of them. Four studies presented analyses derived from the CRIC (Chronic Renal Insufficiency Cohort) study [37, 43–45]. The median participants’ age was 57 years, while 55.5% of patients were males. In the majority of studies, sST2 levels were measured with enzyme linked immunosorbent assays (ELISA). The most commonly applied ELISA assay was the Presage ST2 assay (Critical Diagnostics, New York, N.Y., USA), while a different assay was used by Obokata et al*.* [51] (Medical & Biological Laboratories, Woburn, MA). The risk of bias was judged to be low in 7 and moderate in 14 studies (Table 2). Specifically, concerns of confounding were raised in 10 studies due to potentially inadequate adjustment for important covariates, while selection bias could not be safely excluded in 7 studies due to lack of information regarding the possibility of participant selection based on their characteristics. Additionally, a moderate risk of bias was recognized in the domain of selection of the reported result in 6 studies due to limited available information concerning the analysis plan and the reporting of effect estimates.

**Table 1:** Methodological characteristics of the included studies

Study	Country	Study design	Sample size	Type of population	sST2 assay	Age (years)^†^	Male sex (%)	Hypertension (%)	Diabetes mellitus (%)	BMI (kg/m^2^)^†^	eGFR (ml/min/1.73 m^2^)^†^	Dialysis vintage (months)	CVD (%)
2022; Hammer	Germany	PC	1,196	Hemodialysis	ELISA	66.2	54.2	90	100	27.6	< 15	8.3	29.5
2022; Zhou	China	RC	111	Hemodialysis	NR	58.8	59.5	NR	NR	NR	< 15	44.2	16.2
2022; Lidgard	USA	PC	2,560	eGFR < 70 ml/min/1.73 m^2^	ELISA	56	51	NR	43	32	45.7	NA	NR
2021^b^; Kim	South Korea	PC	296	Hemodialysis	ELISA	57	53	86.5	45.6	22.5	< 15	48.5	23.6
2021^a^; Kim	South Korea	PC	352	Pre-dialysis CKD	ELISA	54	59.9	72.7	28.1	24.7	59	NA	18.5
2020; Choi	South Korea	PC	74	Peritoneal dialysis	ELISA	53.9	63.5	NR	28.4	23.1	< 15	30	13.5
2020; Wang	USA	PC	3,664	eGFR < 70 ml/min/1.73 m^2^	ELISA	57.8	54	NR	49	32.1	44.3	NA	33
2019; Feldreich	Sweden	PC	183	Hemodialysis	Multiplex kit	63	45	NR	25	NR	< 15	44	19
2019^b^; Bansal	USA	PC	3,314	eGFR < 70 ml/min/1.73 m^2^	ELISA	57.5	54	NR	47	31.9	44.7	NA	26
2019^a^; Bansal	USA	PC	3,664	eGFR < 70 ml/min/1.73 m^2^	ELISA	57.8	54.3	NR	48.7	32.1	44.3	NA	32.9
2018; Homsak	Slovenia	PC	123	Hemodiafiltration	ELISA	66	58.5	91	36.6	NR	< 15	50	26
2018; Seo	South Korea	RC	182	Hemodialysis	ELISA	61.3	58.2	80.8	56	23.8	< 15	NR	NR
2018; Tuegel	USA	PC	883	Pre-dialysis CKD	ELISA	57	56	87	43	31.7	49	NA	40
2018; Plawecki	France	RC	218	Pre-dialysis CKD	ELISA	68.3	64	NR	NR	NR	37	NA	
2018; Alam	USA	PC	841	Pre-dialysis CKD	ELISA	57	55	86	42	31.7	51	NA	36
2017; Zhang	China	PC	414	Hemodialysis	ELISA	61.8	61.6	94	22.9	22	< 15	35	9.2
2017; Gungor	Turkey	PC	238	Pre-dialysis CKD	ELISA	49.6	NR	22.7	25.2	NR	47.5	NA	13
2016; Obokata	Japan	PC	423	Hemodialysis	ELISA	66	68.8	84.6	46.6	22.7	< 15	5.8	16.5
2016; Bansal	USA	PC	2,763	Pre-dialysis CKD	ELISA	72	37	58	16	26.7	NR	NA	16
2013; Bayes-Genis	Spain	PC	534	CKD stage 3–4	ELISA	70.4	71.8	61.1	35.7	26.9	51.2	NA	52.7
2013; Ho	USA	PC	2,614	Pre-dialysis CKD	ELISA	57	46	24	7	27.8	87	NA	3

*NR* not reported; *NA* not applicable; *PC* prospective cohort; *RC* retrospective cohort; *BMI* body mass index; *eGFR* estimated glomerular filtration rate; *CVD* cardiovascular disease; *ELISA* enzyme-linked immunosorbent assay; *CKD* chronic kidney disease

^**†**^Median value

**Table 2:** Outcomes of the ROBINS-I evaluation

*Study*	*Bias due to confounding*	*Bias in selection of participants into the study*	*Bias in classification of exposures*	*Bias due to deviations from intended exposures*	*Bias due to missing data*	*Bias in measurement of outcomes*	*Bias in selection of the reported result*	*Overall bias*
2022; Hammer	Low	Low	Low	Low	Low	Low	Low	Low
2022; Zhou	Moderate	Moderate	NI	Low	NI	Low	Low	Moderate
2022; Lidgard	Low	Low	Low	Low	Low	Low	Low	Low
2021; Kim	Low	Low	Low	Low	Low	Low	Moderate	Moderate
2021; Kim	Low	Moderate	Low	Low	Low	Low	Low	Moderate
2020; Choi	Moderate	Moderate	Low	Low	Low	Low	Low	Moderate
2020; Wang	Low	Low	Low	Low	Low	Low	Low	Low
2019; Feldreich	Moderate	Low	Low	Low	Low	Low	Low	Moderate
2019^b^; Bansal	Low	Low	Low	Low	Low	Low	Low	Low
2019^a^; Bansal	Low	Low	Low	Low	Low	Low	Low	Low
2018; Homsak	Moderate	Moderate	Low	Low	Low	Low	Moderate	Moderate
2018; Seo	Moderate	Low	Low	Low	Low	Low	Low	Moderate
2018; Tuegel	Low	Low	Low	Low	Low	Low	Low	Low
2018; Plawecki	Moderate	Moderate	Low	Low	Low	Low	Moderate	Moderate
2018; Alam	Low	Low	Low	Low	Low	Low	Low	Low
2017; Zhang	Moderate	Low	Low	Low	Low	Low	Low	Moderate
2017; Gungor	Moderate	Low	Low	Low	Low	Low	Moderate	Moderate
2016; Obokata	Moderate	Low	Low	Low	Low	Low	Moderate	Moderate
2016; Bansal	Low	Low	Low	Low	Low	Low	Moderate	Moderate
2013; Bayes-Genis	Moderate	Moderate	Low	Low	Low	Low	Low	Moderate
2013; Ho	Low	Moderate	Low	Low	Low	Low	Low	Moderate

*NI* no information

### Pre-dialysis population

#### All-cause mortality

The association of sST2 levels with overall survival of pre-dialysis chronic kidney disease patients was evaluated in 3 studies (Table 3). All studies showed that increasing sST2 was linked to a significantly higher risk of all-cause mortality. Specifically, the CRIC study including 3,664 participants with mildly to moderately impaired renal function (median eGFR: 44.3 ml/min/1.73 m^2^) suggested that high sST2 values were associated with a significantly elevated mortality risk when sST2 was treated both as continuous and binary variable [44]. Similar outcomes were obtained by the pooled analysis of the SKS (Seattle Kidney Study) and C-PROBE (Clinical Phenotyping and Resource Biobank Study) cohorts (883 patients, HR per standard deviation increase: 1.36, 95% CI: 1.17 to 1.58) [41], as well as by a prospective cohort of 534 patients with stage 3–4 chronic kidney disease (HR per increase by 10 ng/ml: 1.22, 95% CI: 1.06 to 1.42) [46].

**Table 3:** Association of sST2 with mortality and adverse cardiovascular outcomes

Study	Mortality	MACE	Cardiovascular mortality	Death or MACE	Heart failure
Dialysis					
2022; Hammer	*sST2 20.1–25 ng/ml HR:* 1.12 (0.87–1.45)	–	*sST2 20.1–25 ng/ml HR:* 1.26 (0.83–1.91)	–	–
	*sST2 25.1–32.6 ng/ml HR:* 1.64 (1.25–2.16)*		*sST2 25.1–32.6 ng/ml HR:* 1.87 (1.55–3.39)*		
	*sST2* > *32.6 ng/ml HR*: 2.06 (1.61–2.61)*		*sST2* > *32.6 ng/ml HR*: 2.29 (1.55–3.39)*		
2022; Zhou	–	–	–	–	*Per 1 sST2 unit HR*: 1.03 (1.01–1.05)*
2021; Kim	*Per 1 log-sST2 unit HR*: 1.60 (1.02–2.48)*	*Per 1 log-sST2 unit HR*: 0.99 (0.75–1.32)	–	–	–
2020; Choi	*Per 1 sST2 SD HR*: 1.94 (1.12–3.36)*	*Per 1 sST2 SD HR*: 1.63 (1.07–2.48)*	–	–	–
	*sST2* ≥ *70.9 ng/ml HR*: 10.14 (2.16–47.73)*	*sST2* ≥ *70.9 mg/dl HR*: 3.93 (1.43–10.92)*			
2019; Feldreich	–	–	*Per 1 sST2 SD HR*: 1.63 (1.13–2.35)*	–	–
2018; Homsak	*Per 1 sST2 unit HR*: 1.02 (1.01–1.02)*	–	*Per 1 sST2 unit HR*: 1.01 (1.01–1.02)*	–	–
	*sST2* > *48 ng/ml HR*: 3.64 (1.61–8.21)*		*sST2* > *44 ng/ml HR*: 2.67 (1.14–7.13)*		
2018; Seo	*sST2* ≥ *59.5 mg/dl HR*: 2.62 (1.11–6.24)*	–	*sST2* ≥ *59.5 mg/dl HR*: 2.68 (0.96–7.53)	*Per 1 sST2 unit HR:* 1.008 (1.003–1.013)*	–
				*sST2* ≥ *59.5 mg/dl HR*: 2.33 (1.12–4.87)*	
2017; Zhang	*Per 1 log-sST2 SD:* 1.31 (1.00–1.72)*	–	–	–	–
2016; Obokata	*Per 1 log-sST2 unit HR*: 10.6 (4.98–22.5)*	–	–	*Per 1 log-sST2 unit HR*: 10.6 (4.98–22.5)*	–
	*sST2 0.237–0.299 ng/ml HR*: 1.12 (0.43–2.91)			*sST2 0.237–0.299 ng/ml HR*: 0.93 (0.46–1.88)	
	*sST2* ≥ *0.299 ng/ml HR*: 4.15 (1.91–9.03)*			*sST2* ≥ *0.299 ng/ml HR*: 3.21 (1.82–5.66)*	
Pre-dialysis CKD					
2022; Lidgard	–	*Per 1 log-sST2 SD HR*: 1.19 (1.04–1.36)*	–	–	–
2021; Kim	–	–	–	*Per 1 log-sST2 unit HR*: 2.11 (1.19–3.74)*	–
2020; Wang	*Per 1 log-sST2 unit HR*: 1.16 (1.07–1.25)*	–	–	–	–
	*sST2 10.6–13.6 ng/ml HR*: 1.07 (0.84–1.41)				
	*sST2 13.7–17.2 ng/ml HR*: 1.12 (0.88–1.43)				
	*sST2 17.3–22.9 ng/ml HR*: 1.38 (1.10–1.74)*				
	*sST2* > *22.6 ng/ml HR*: 1.32 (1.04–1.68)*				
2019; Bansal	–	–	–	–	*Per 1 log-sST2 SD HR*: 1.20 (1.05–1.36)*
					*sST2 10.6–13.6 ng/ml HR*: 0.97 (0.67–1.41)
					*sST2 13.7–17.1 ng/ml HR*: 1.29 (0.91–1.83)
					*sST2 17.2–22.6 ng/ml HR*: 1.53 (1.08–2.16)*
					*sST2* > *22.6 ng/ml HR*: 1.63 (1.16–2.30)*
2018; Tuegel	*Per 1 sST2 SD unit HR*: 1.36 (1.17–1.58)*	*Per 1 sST2 SD unit HR*: 1.16 (0.75–1.78)	–	–	*Per 1 sST2 unit HR*: 1.22 (0.94–1.60)
2018; Plawecki	–	–	–	*Per 1 log-sST2 unit HR*: 2.84 (0.53–15.13)	–
2017; Gungor	–	–	–	*Per 1 sST2 unit HR*: 1.002 (1.00–1.003)*	
2013; Bayes-Genis	*Per sST2 increase by 10 ng/ml*: 1.22 (1.06–1.42)*				

*HR* hazard ratio; *SD* standard deviation; *MACE* major adverse cardiovascular events; *CKD* chronic kidney disease

*p value < 0.05

#### Cardiovascular disease

The endpoint of MACE was assessed in 2 studies with mixed results (Table 3). The analysis of the CRIC study (2,560 participants) showed that higher sST2 levels were linked to a significantly increased risk of a 3-point MACE occurrence, defined as myocardial infarction, stroke or peripheral artery disease (HR per log-standard deviation increase: 1.19, 95% CI: 1.04 to 1.36) [43]. On the contrary, no significant association of sST2 with the composite of myocardial infarction or stroke (HR: 1.16, 95% CI: 0.75 to 1.78) by Tuegel et al. [41] (883 participants). In addition, two studies [38, 40] (352 and 238 patients, respectively) proposed that increasing sST2 levels were associated with higher risk of the composite endpoint of death or MACE, although this effect was not confirmed by another study including 218 patients [42]. The risk of incident heart failure was evaluated by 2 studies; although Tuegel et al. [41] showed no significant association with sST2, the analysis of the CRIC study [45] suggested a significant link between increasing sST2 levels and incident heart failure (HR per log-standard deviation: 1.20, 95% CI: 1.05 to 1.36).

#### Kidney disease progression

**Fig. 2:**
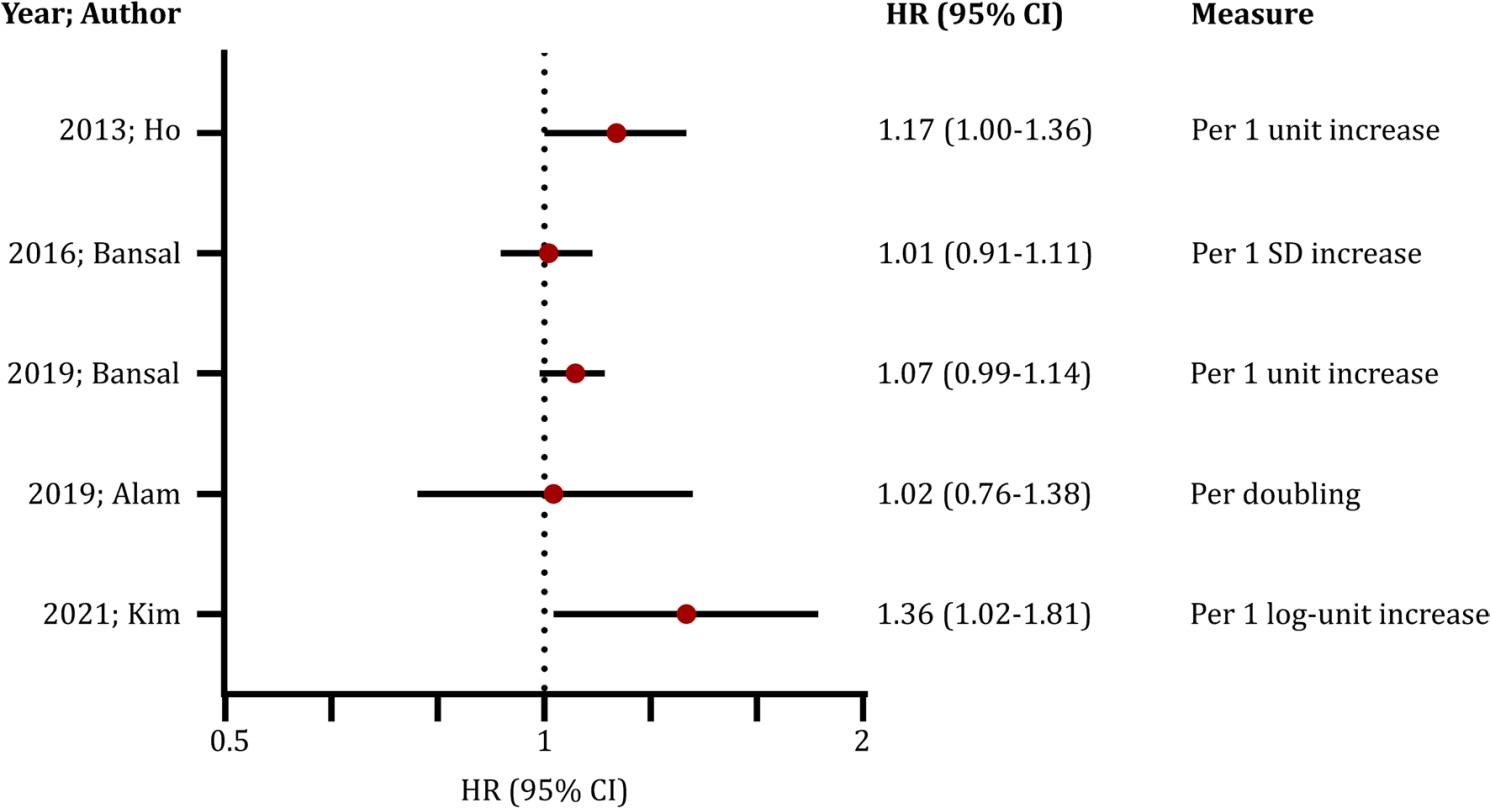
Outcomes of studies evaluating the association of sST2 levels with the risk of kidney disease progression. *HR* hazard ratio; *CI* confidence intervals

The association of circulating sST2 with kidney disease progression was examined in 5 studies (Table 4), reporting conflicting results. An analysis of the Framingham Heart Study offspring cohort [39] proposed that increasing sST2 is marginally associated with rapid kidney function decline (≥ 3 ml/min/1.73 m^2^ per year – HR: 1.17, 95% CI: 1.00–1.36, moderate risk of bias). This outcome was corroborated by a cohort of 352 patients [38], indicating that high sST2 values are associated with an elevated risk of an eGFR reduction more than 50% or requirement of renal replacement therapy (HR per log-unit increase: 1.36, 95% CI: 1.02 to 1.81, moderate risk of bias). However, no significant association between elevated sST2 and subsequent kidney disease progression was observed in three large prospective cohort studies at low risk of bias (SKS/C-PROBE, CRIC and Cardiovascular Health Study) [37, 49, 57] (Fig. 2).

**Table 4:** Association of sST2 levels with kidney disease progression

Study	Definition of kidney disease progression	Outcome
2021; Kim	≥ 50% eGFR reduction or RRT	*Per 1 log-sST2 unit HR*: 1.36 (1.02–1.81)*
2019; Alam	eGFR < 15 ml/min/1.73 m^2^ or RRT	*Per sST2 doubling HR*: 1.02 (0.76–1.38)
		*sST2 20.52–26 ng/ml HR*:1.38 (0.84–2.27)
		*sST2 26.01–34.14 ng/ml HR*: 1.36 (0.81–2.29)
		*sST2* > *34.14 ng/ml HR*: 1.54 (0.92–2.58)
2019; Bansal	≥ 50% eGFR reduction or RRT	*Per 1 sST2 unit HR*: 1.07 (0.99–1.14)
		*sST2 10.6–13.6 ng/ml HR*: 0.97 (0.78–1.21)
		*sST2 13.7–17.2 ng/ml HR:* 0.95 (0.76–1.19)
		*sST2 17.3–22.9 ng/ml HR*: 1.02 (0.82–1.27)
		*sST2* > *22.9 ng/ml HR*: 1.19 (0.95–1.50)
2016; Bansal	eGFR decline ≥ 30%	*Per 1 sST2 SD HR*: 1.01 (0.91–1.11)
		*sST2 18.84–23.62 ng/ml HR*: 1.08 (0.86–1.36)
		*sST2 23.63–29.72 ng/ml HR*: 1.03 (0.81–1.30)
		*sST2* > *29.72 ng/ml HR*: 1.00 (0.76–1.30)
2013; Ho	eGFR decline ≥ 3 ml/min/1.73 m^2^ per year	*Per 1 sST2 unit HR*: 1.17 (1.00–1.36)*

*HR* hazard ratio; *SD* standard deviation; *eGFR* estimated glomerular filtration rate; *RRT* renal replacement therapy

^*^p value < 0.05

### Dialysis population

#### All-cause mortality

**Fig. 3:**
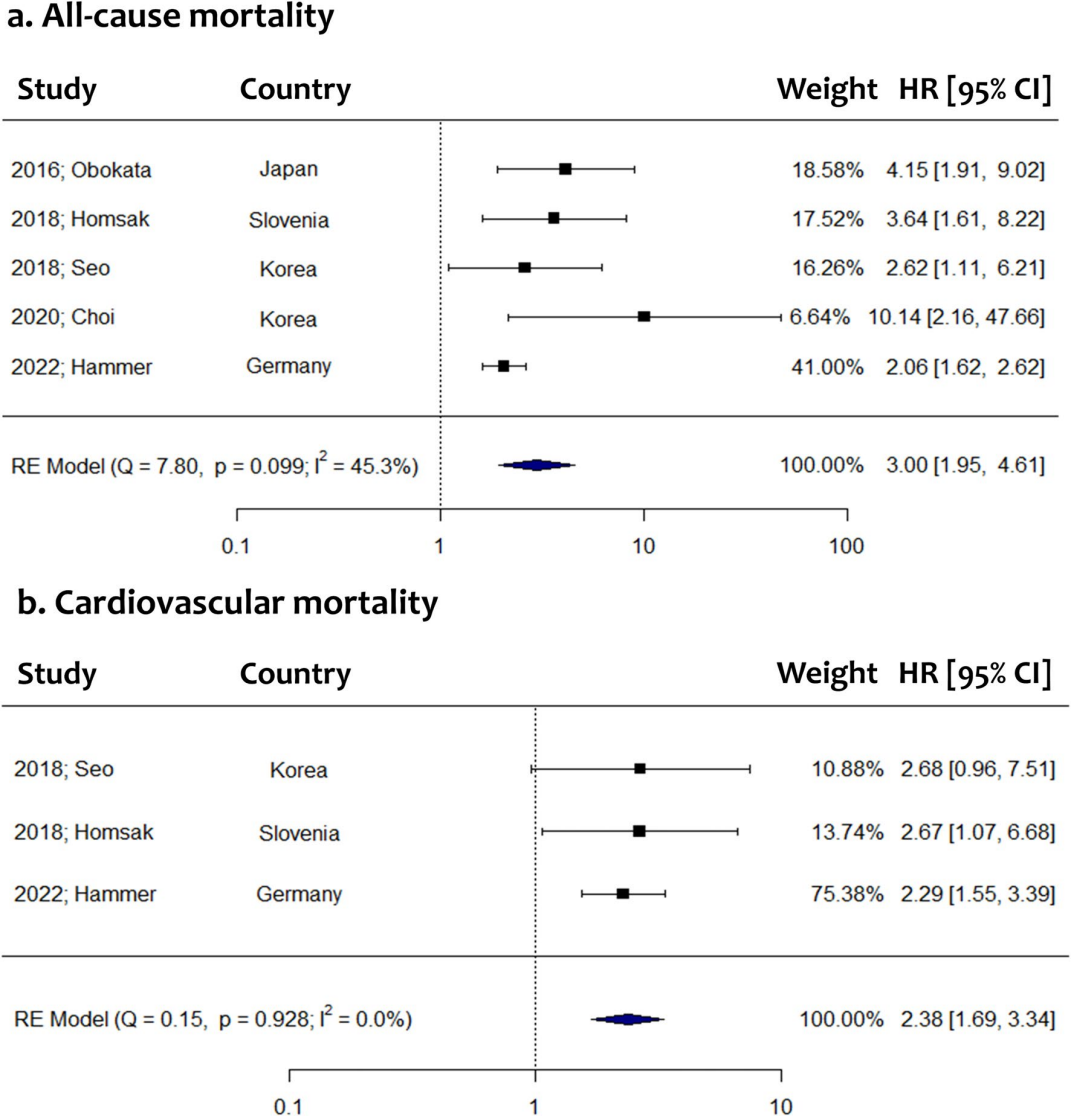
Forest plots comparing the highest to the lowest circulating sST2 categories in regards to all-cause mortality (**a**) and cardiovascular mortality (**b**) among dialysis patients. *RE* random-effects; *HR* hazard ratio; *CI* confidence intervals

**Fig. 4:**
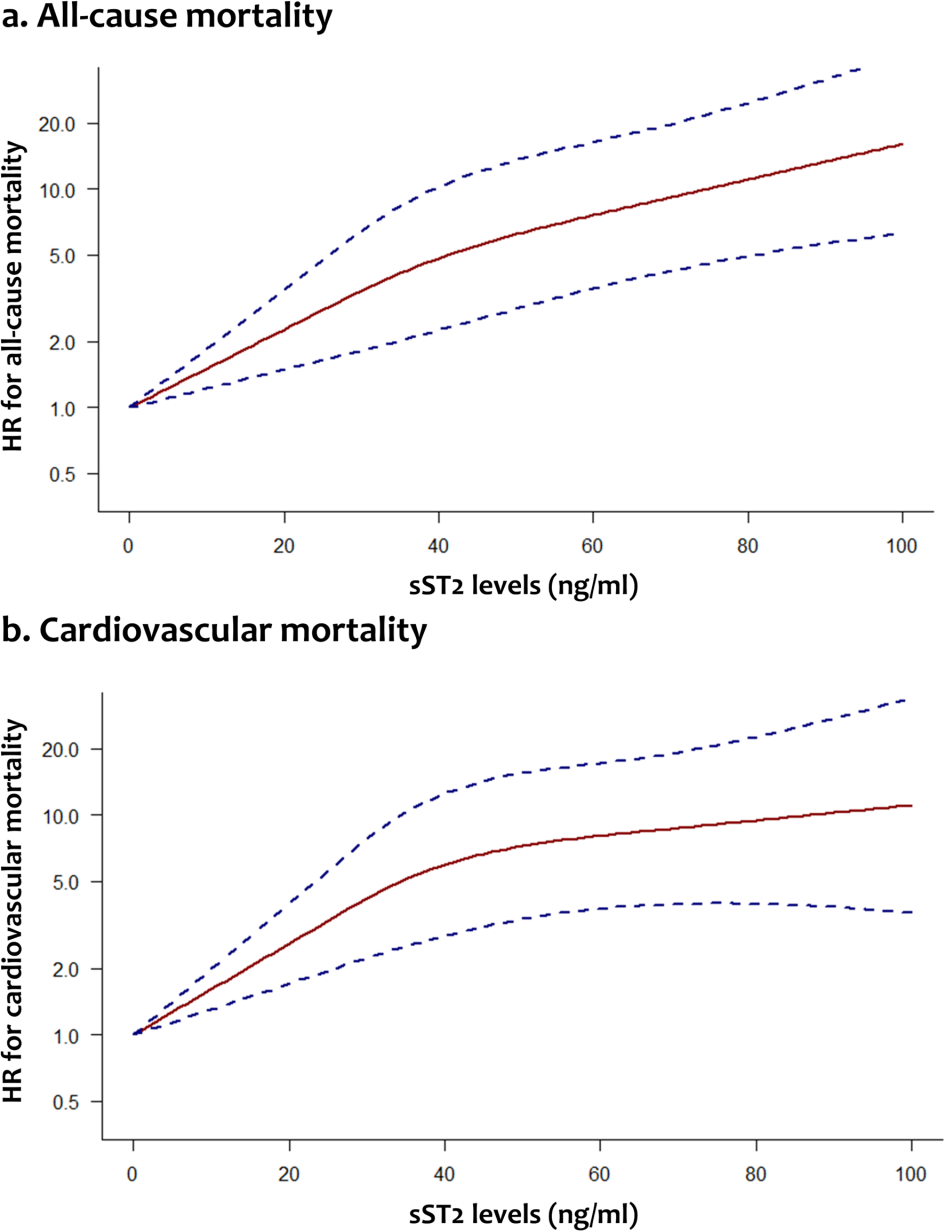
Relationship between circulating sST2 levels and risk of all-cause mortality (**a**) and cardiovascular mortality (**b**) among dialysis patients. Dashed lines represent 95% confidence intervals. *HR* hazard ratio

The association of sST2 with overall survival of dialysis patients was assessed in 7 studies (Table 3). All studies suggested that elevated sST2 values are associated with a significantly higher risk of all-cause mortality when sST2 was treated either as a continuous or a binary variable. Conventional meta-analysis of studies that introduced thresholds proposed that the highest category of sST2 is associated with significantly increased mortality risk (5 studies, HR: 3.00, 95% CI: 1.95 to 4.61). A similar outcome was obtained using the Knapp-Hartung adjustment (HR: 3.00, 95% CI: 1.65 to 5.45) (Fig. 3). The statistical heterogeneity was estimated to be moderate (*I*^*2*^: 45.3%), while the 95% prediction intervals ranged from 1.40 to 6.41. The dose–response meta-analysis included 4 studies and confirmed that increasing sST2 values are associated with a significantly higher mortality risk (*χ*^*2*^: 34.65, *p value* < 0.001) (Fig. 4). Compared to a reference sST2 value of 10 ng/ml, a significantly elevated mortality risk was estimated for sST2 levels of 20 ng/ml (HR: 1.51, 95% CI: 1.22 to 1.86), 40 ng/ml (HR: 3.19, 95% CI: 1.85 to 5.49), 60 ng/ml (HR: 5.02, 95% CI: 2.82 to 8.94), 80 ng/ml (HR: 7.30, 95% CI: 3.75 to 14.19) or 100 ng/ml (HR: 10.61, 95% CI: 4.50 to 25.01).

#### Cardiovascular mortality

The association of circulating sST2 with cardiovascular mortality of dialysis patients was examined in 4 studies (Table 3). Three of them reported a significant association between high sST2 and increased cardiovascular mortality risk. Meta-analysis indicated that sST2 in the highest category is associated with a significantly elevated risk of cardiovascular mortality (3 studies, HR: 2.38, 95% CI: 1.69 to 3.34) (Fig. 3). Using the Knapp-Hartung adjustment led to a similar estimate (HR: 2.38, 95% CI: 1.94 to 2.92). No statistical heterogeneity was observed (*I*^*2*^: 0%) and thus the 95% prediction interval was identical to the confidence interval. The dose–response meta-analysis also suggested that increasing sST2 is significantly associated with higher cardiovascular mortality risk (*χ*^*2*^: 29.14, *p value* < 0.001) (Fig. 4). Compared to a reference sST2 value of 10 ng/ml, a significantly increased cardiovascular mortality risk was estimated for sST2 levels of 20 ng/ml (HR: 1.61, 95% CI: 1.31 to 1.97), 40 ng/ml (HR: 3.68, 95% CI: 2.16 to 6.28), 60 ng/ml (HR: 4.97, 95% CI: 2.77 to 8.92), 80 ng/ml (HR: 5.84, 95% CI: 2.70 to 12.65) or 100 ng/ml (HR: 6.86, 95% CI: 2.31 to 20.27).

#### Cardiovascular disease

Two studies have evaluated the association between sST2 and MACE (Table 3). In particular, Choi et al*.* [56] proposed that among 74 peritoneal dialysis patients, high sST2 was associated with a significantly increased risk of MACE, defined by the presence of acute coronary syndrome, stable angina requiring revascularization, congestive heart failure or cerebrovascular accident. On the contrary, a prospective cohort study of 296 hemodialysis patients supported no significant association between sST2 and MACE risk (HR per log-unit increase: 0.99, 95% CI: 0.75 to 1.32) [52]. The composite outcome of death or MACE was examined by 2 studies. Specifically, a prospective cohort study including 423 hemodialysis patients suggested that high circulating sST2 was associated with a significantly increased risk of mortality, myocardial infarction, stroke or hospitalization for heart failure (HR per log-unit increase: 10.6, 95% CI: 4.98 to 22.5) [51]. In addition, Seo et al*.* [50] showed that among 182 hemodialysis patients, elevated sST2 was linked to a significantly higher risk of death or MACE, although the composite outcome was mainly driven by all-cause mortality rather than cardiovascular events. The endpoint of heart failure was assessed by one study (111 participants), indicating an elevated risk of incident heart failure in hemodialysis patients with high sST2 values (HR: 1.03, 95% CI: 1.01 to 1.05) [47].

## Discussion

The present systematic review collected the available evidence coming from 21 cohort studies and 15,100 patients, evaluating the prognostic role of sST2 in chronic kidney disease. The qualitative synthesis of studies on pre-dialysis patients suggested that high circulating sST2 may be associated with worse survival rates, while data regarding its association with the occurrence of cardiovascular events and kidney disease progression are currently conflicting. More robust evidence exists on the dialysis population in which dose–response meta-analysis indicated that increasing circulating sST2 values are associated with an elevated risk of both all-cause and cardiovascular mortality in a log-linear fashion. No specific prognostic role could be supported for sST2 in regards to cardiovascular events among dialysis patients since limited data with mixed results were available.

The findings of this study corroborate prior research demonstrating the role of sST2 in the prediction of patient survival. A growing body of evidence suggests that high sST2 levels are linked to increased mortality in individuals with pre-existing cardiovascular disease [58]. The pathophysiology of this observation may explained by the attenuation of interleukin-33/ST2 cardioprotective properties, leading to maladaptive myocardial hypertrophy and fibrosis [59]. In addition, circulating sST2 has been shown to be predictive of mortality in various inflammatory conditions, such as HIV (human immunodeficiency virus) infection [60], sepsis [61] and acute pancreatitis [62]. The role of sST2 in inflammatory processes is mainly based on the regulation of innate and adaptive immunity via the inhibition of the interleukin-33-mediated release of Th2 cytokines, such as interleukin-4, interleukin-5 and interleukin-13 [63]. In this context, circulating sST2 has been shown to correlate with serum high-sensitivity C-reactive protein in chronic kidney disease patients [40], as well as to effectively predict infection-related mortality in the dialysis population [54].

Despite the prognostic role of sST2 in regards to overall and cardiovascular mortality, current evidence as assessed in this systematic review suggests a less clear association with cardiovascular events. This finding is in line with previous research proposing no clear or significant link between circulating sST2 and MACE in the general population [64, 65]. Similarly, the KAROLA study [66] has indicated that among patients with stable coronary heart disease, sST2 levels were prognostic of all-cause and cardiovascular mortality but not of non-fatal cardiovascular events. Regarding echocardiographic parameters, circulating sST2 has been associated with left ventricular relative wall thickness and concentric hypertrophy among dialysis patients [29], although this was not confirmed for pre-dialysis individuals in the CRIC cohort [33]. It should be also noted that the potential link between sST2 and incident atrial fibrillation has been also examined in the CRIC study, proposing a modest but inconsistent association in the categorical analyses [30].

The existing evidence regarding the association of sST2 with renal function is mixed. Early studies have proposed that sST2 is not affected by the presence of chronic kidney disease [67, 68]. However, a weak to modest negative correlation of sST2 levels with eGFR has been suggested by recent studies in the field, especially when patients with advanced renal dysfunction were evaluated [34, 46, 52]. The findings of this systematic review could not ascertain a potential predictive role of sST2 levels in regards to kidney disease progression since negative outcomes were derived from the outcomes of three large prospective cohort studies (SKS/C-PROBE, CRIC and Cardiovascular Health Study) [37, 49, 57].

The present study has several strengths. Literature has been systematically searched by applying a comprehensive algorithm in 5 different databases, without applying any date restrictions. The risk of bias was critically assessed, allowing a realistic appraisal of study limitations. This systematic review extends the outcomes of previous ones in the field [69, 70] by including a significantly larger number of studies of both pre-dialysis and dialysis patients and by implementing a strict statistical methodology that avoids the pooling of studies reporting different effect measures. Apart from conventional meta-analysis, dose–response meta-analysis was also conducted, allowing the definition of the exact relationship between sST2 and mortality risk across the whole range of the biomarker levels.

On the other hand, the interpretation of outcomes is limited by the remarkable inter-study heterogeneity, especially concerning MACE and kidney disease progression definitions, as well as outcome reporting. As a result, a quantitative pooling of studies was feasible only for dialysis mortality and all other endpoints were qualitatively evaluated. In addition, the small number of studies per outcome precluded the conduct of subgroup analyses, as well as the assessment of publication bias. It should be also acknowledged that only 1 study included peritoneal dialysis patients and thus the generalizability of outcomes in this population remains limited. Preliminary evidence has indicated that peritoneal dialysis may be linked to lower circulating sST2 levels compared to hemodialysis [71], although the exact prognostic efficacy of the biomarker in regards to mortality and cardiovascular events warrants further exploration among peritoneal dialysis patients.

The present study provides evidence supporting the promising role of circulating sST2 as a predictor of survival in patients undergoing maintenance dialysis. Due to is large molecular weight (37 kDa), its levels are not affected by hemodialysis, even with high-flux dialyzers [72]. The potential clinical utility of circulating sST2 as a biomarker is also reinforced by its low biological variation, while its low index of individuality renders it suitable for serial testing to identify changes over time that would potentially indicate cardiovascular disease progression and increased mortality risk [73]. Future large-scale cohort studies are needed to define the exact applicability of sST2 in clinical practice, by further exploring its potential association with cardiovascular events, incident heart failure, as well as infectious complications. Circulating sST2 may be evaluated in conjunction with both traditional risk factors and novel biomarkers, such as natriuretic peptides, galectin-3 and growth/differentiation factor-5, aiming to construct combined models that would achieve optimal prognostic efficacy.

In conclusion, the present systematic review and meta-analysis suggested that sST2 is associated with dialysis survival, presenting a log-linear relationship with both all-cause and cardiovascular mortality risk. Among non-dialysis chronic kidney disease patients, limited evidence suggests that high circulating sST2 may be also linked to an elevated mortality risk. Conflicting data are currently available concerning the association of sST2 with cardiovascular events and thus further large-scale studies are needed in order to reach firm conclusions about its role in cardiovascular prediction among individuals with chronic kidney disease.

## Data Availability

Data are available from the corresponding author upon request.
